# Natural Silkworm Cocoon Composites with High Strength and Stiffness Constructed in Confined Cocooning Space

**DOI:** 10.3390/polym10111214

**Published:** 2018-10-31

**Authors:** Lan Cheng, Xiaoling Tong, Zhi Li, Zulan Liu, Huiming Huang, Hongping Zhao, Fangyin Dai

**Affiliations:** 1 State Key Laboratory of Silkworm Genome Biology, Key Laboratory of Sericultural Biology and Genetic Breeding, Ministry of Agriculture, College of Biotechnology, Southwest University, Chongqing 400715, China; chenglan2018@swu.edu.cn (L.C.); xltong@swu.edu.cn (X.T.); 2 Chongqing Engineering Research Center of Biomaterial Fiber and Modern Textile, College of Textile and Garment, Southwest University, Chongqing 400715, China; tclizhi@swu.edu.cn (Z.L.); 13658349461@163.com (Z.L.); 3 Institute of Biomechanics and Medical Engineering, Tsinghua University, Beijing 100084, China; hhm207@163.com

**Keywords:** silkworm cocoons, dense structure, porosity, robust fiber network, mechanical properties

## Abstract

In this study, using round paper tubes (PTs) and rectangular cardboard boxes (CBs) as external constraints to control the size of the cocooning space, we fabricated a series of modified silkworm cocoons (PT cocoons and CB cocoons). Their microstructures, morphologies, compositions, and mechanical properties were characterized and compared with normal silkworm cocoons. These two kinds of modified silkworm cocoons exhibit dense and homogeneous layer structures. Tensile test results indicate that above a size limit of cocooning space, their tensile strengths, Young’s moduli, and strain energy densities increase with the decrease in cocooning space. Especially in comparison with the normal cocoons, the tensile strength and Young’s modulus of the PT-14 cocoon increase by 44% and 100%, respectively. Meanwhile, PT cocoons and CB cocoons, except PT-12, also possess better peeling resistance than normal cocoons. Owing to the dense structure and low porosity, the modified cocoons form robust fiber networks that result in high strength and toughness. This study provides a green and efficient method to fabricate mechanically enhanced silkworm cocoons with special shapes and dense layer structures. The method can be easily subjected to further modification processes and has potential applications in the production of high-performance green cocoon composites and biomimetic materials.

## 1. Introduction

Silkworm cocoons are biological structural materials constructed by silkworm larvae and provide protection from the natural environment, parasitism, or predators of silkworm pupae [1,2,3,4,5]. The silkworm cocoon could be used as protective materials, sorbent materials, gas filters, and biosensors considering its porous hierarchical structure, good impact resistance [6], sorption capacity [7], temperature- and humidity-dependent electrical properties [8], and photoelectrical properties [9,10,11,12]. Increasing attention has been paid to modifying the microstructure and improving the properties of silkworm cocoons without damaging their biological structures for further applications to the field of composites and biomimetic materials [13,14,15,16,17,18,19].

Cocooning behavior is an important factor that affects the properties of silkworm cocoons. When cocooning begins, a silkworm larva starts to construct a widely spaced supporting network by stretching and turning its segmented body to search for potential attachment substrates and constructs a framework. Then, the larva turns its body around by swinging its head in figure-of-eight motions to overlap the silk fibers, ultimately producing a cocoon to protect the pupa from predators’ attacks. The construction strategy of silkworm larvae depends on the silkworm species and the natural environment, resulting in diverse cocoon sizes, microstructures, morphologies, and mechanical properties [20,21,22,23,24]. On one hand, the relationship between the cocooning behaviors of a silkworm larva and the silkworm cocoon’s characteristics has been systematically revealed and visually examined by 3D computer graphics software [25,26,27,28,29,30]. On the other hand, the cocooning environment, such as the size of the cocooning space, can also affect the cocoon’s characteristics, including the shape [23] and the mechanical properties. Silkworms have evolved by the process of natural selection over millions of years to construct its corresponding shield housing for survival under a complex confined cocooning space. However, to date, little research has been conducted on the relationship between the confined cocooning space and the mechanical properties of silkworm cocoons.

Owing to its special biological structure, the silkworm cocoon can be regarded as a nonwoven fiber composite and has inspired people to design and fabricate advanced engineering materials [31,32,33]. However, silkworm cocoons are difficult to be directly used for manufacturing artificial composites due to their irregular shape, as well as the inhomogeneous cocoon layers [1,5]. Meanwhile, the *Bombyx mori* cocoon has been categorized as a “weak” cocoon with high porosity and relatively lower mechanical properties compared to some wild silkworm cocoons [3]. The study by Guan et al. [18] on the structure and properties of different kinds of cocoons revealed that the mechanical properties of cocoons are determined by fiber networks and fiber properties. A prerequisite of the cocoon structure is a robust fiber network, in which fibers with good mechanical properties play a part [18]. Tremendous efforts have been made to investigate the mechanical properties of the silk fibers [34,35] and to fabricate enhanced silk fibers using various methods [36,37,38,39,40,41]. However, there is a lack of studies on the preparation of enhanced silkworm cocoons, especially in a practical and green way, without damaging their special biological structures. Taking advantage of the ability of silkworm larvae to construct their shield housing under complex external constraints, silkworm cocoons with controllable shapes and strengthened fiber networks could be obtained.

In this study, we chose round paper tubes (PTs) and rectangular cardboard boxes (CBs) as the spinning regions for silkworm larvae in the lab when cocooning begins. The sizes of the constructed spaces were controlled by the spatial alternation of tube diameter for the PTs and the box width for the CBs to obtain a series of modified silkworm cocoons, named PT cocoons and CB cocoons, respectively. Normal silkworm cocoons (NCs) were constructed with common paper cocooning frames for silkworm larvae. The morphologies, microstructures, sericin contents, and mechanical properties of these modified silkworm cocoons were experimentally characterized and subsequently compared with those of normal silkworm cocoons. Finally, the relationship between the mechanical properties and the microstructures of the silkworm cocoons was further investigated. The results revealed that enhanced mechanical properties of silkworm cocoons constructed in a confined space were obtained, especially due to the dense cocoon microstructure. This study could help to develop strengthened cocoon materials using highly efficient strategies for potential applications in the field of composite engineering and biomimetic science.

## 2. Materials and Methods

### 2.1. Fabrication of Normal and Modified Cocoons

**Figure 1:**
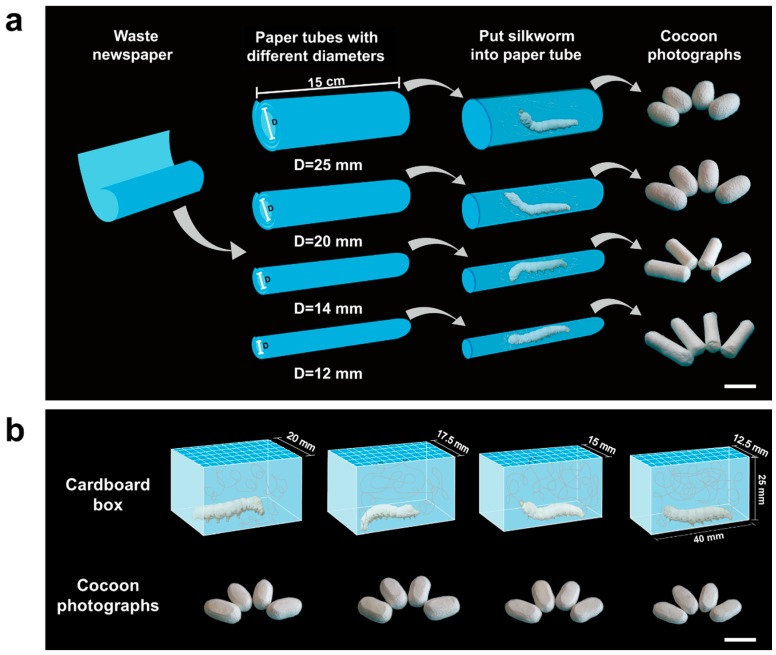
Schematic illustrations for the fabrication processes, and photographs of each type of modified silkworm cocoon: (**a**) paper tube (PT) cocoons and (**b**) cardboard box (CB) cocoons. Scale bar for silkworm cocoons is 2 cm.

*Bombyx mori* silkworms (Chinese strain *Xiafang* × *Qiubai*) were obtained from the State Key Laboratory of Silkworm Genome Biology (Chongqing, China). They were reared under standard conditions and fed with fresh mulberry leaves until they started spinning. Then, each silkworm larva was put into specific PTs with different diameters (25, 20, 14, and 12 mm) or CBs with different widths (20, 17.5, 15, and 12.5 mm) until the cocooning completed. Corresponding modified silkworm cocoons in special shapes were obtained, and the detailed procedure can be seen in Figure 1. The silkworm cocoons produced in the paper tubes with a diameter of 25 mm was labeled as the PT-25 cocoon. Similar labels were applied to the other groups. NCs obtained from normal spinning tools were set as the control group. Twenty cocoons from each group were collected, and their sizes were measured by vernier caliper. Then, the cocoon shell specimens for subsequent measurements were prepared by carefully cutting the cocoons into strips along their longitudinal directions and removing the pupae inside.

### 2.2. Scanning Electron Microscopy (SEM) Observation

Samples were mounted on a platform with a conductive tape backing and then sputter-coated with gold for 2 min. The morphologies of silkworm cocoon layers and cross-sections were examined by SEM (Phenom Pro, Holland, The Netherlands) at an acceleration voltage of 5 kV.

### 2.3. Sericin Concentration

Dried cocoon shell specimens were immersed in 0.5% Na_2_CO_3_ solution at 100 °C for 30 min and then washed with distilled water, followed by drying for 24 h at 40 °C. The concentrations of sericin were calculated by (*w*_0_ − *w*_1_)/*w*_0_, where *w*_0_ and *w*_1_ are the mass of the samples before and after the degumming, respectively [41].

### 2.4. Porosity of Cocoon Shell Specimens

The density (*ρ*_c_) of each cocoon shell specimen was calculated from the weight, area, and thickness of the rectangular specimens. The density (*ρ_f_*) of the silk fibers was set at 1300 kg/m^3^ according to previous studies [18,42]. The porosity (*P*) of the cocoon shell specimens was calculated by the formula $P=1-\rho_{c}/\rho_{f}$. Three samples from each group were used, and all results are given as mean ± standard deviation.

### 2.5. Tensile Tests of Cocoons

Cocoons were cut into strips with a width of 3 mm along the longitudinal direction to prepare the tensile test samples. Quasi-static uniaxial tension tests were performed using an MTS E44-1 kN universal test machine (Shakopee, MN, USA) with a loading rate of 2 mm/min and a gauge length of 10 mm. The thickness of each cocoon shell was measured by vernier caliper. Six samples from each group were examined, and all results are reported as mean ± standard deviation.

### 2.6. Peel Tests of Cocoons

Cocoons were cut into strips with 20 mm lengths and 3 mm widths and peeled artificially from the middle layer to create an interlayer crack of 5 mm. The separated parts of each sample were clamped and peeled at an angle of 90° using the universal test machine with a tensile speed of 2 mm/min and a gauge length of 5 mm. Force-displacement curves for each sample were recorded. Six samples from each group were examined, and all results are reported as mean ± standard deviation.

### 2.7. Theoretical Characterization Models for Porosity

Porosity is an important factor influencing the mechanical performance of silkworm cocoons. Thus, it is necessary to characterize the relationship between porosity and the mechanical properties of silkworm cocoons. Young’s modulus and tensile strength of silkworm cocoons have a similar dependence on the porosity. Based on the studies in [43,44], the Young’s modulus–porosity relationship for silkworm cocoon composites can be defined as:

(1) $$E=E_{0}{\left(1-P/P_{c}\right)}^{n}\;$$

where *E* is the effective Young’s modulus of porous material with porosity *P*, *E*_0_ is Young’s modulus of the silkworm cocoon, *P_c_* is a percolation threshold, i.e., the porosity at which the effective Young’s modulus becomes zero, and *n* is the characteristic exponent. Equation (1) can be directly used to fit the experimentally measured Young’ modulus. In this study, the value of the percolation threshold *P_c_* was set as 1 in the fitting process. This equation should follow the conditions: $E=E_{0}$, at P=0; E=0, at P=1.

The strength–porosity relationship for silkworm cocoons can be characterized by the following widely known inverse proportional mathematic expression [45]:

*σ* = *A*exp(−*kP*) (2)

where *σ* is the strength, *A* is the strength at zero porosity, *P* is the porosity, and *k* is a characteristic exponent. The strengths of the silkworm cocoons at other porosities can be estimated by Equation (2) based on the strength data measured at given porosities.

### 2.8. Statistical Data Analysis

All statistical data are expressed as the mean ± standard deviation. Statistical analyses were conducted using one-way ANOVA as implemented by SPSS statistical software (New York, NY, USA). A *p*-value of <0.05, compared with the control group, was considered to be statistically significant.

## 3. Results and Discussion

### 3.1. Morphology and Microstructure

**Figure 2:**
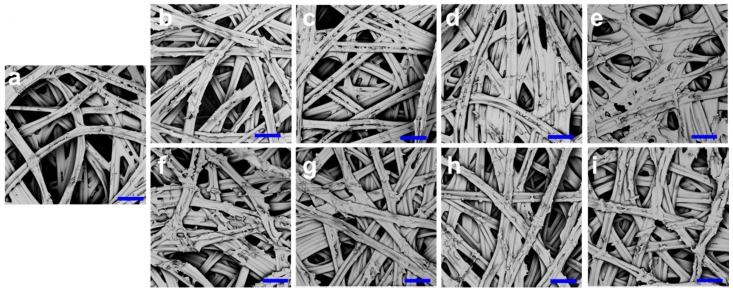
SEM images of cocoon layer structure. (**a**) Normal cocoon; (**b**) PT-25 cocoon; (**c**) PT-20 cocoon; (**d**) PT-14 cocoon; (**e**) PT-12 cocoon; (**f**) CB-20 cocoon; (**g**) CB-17.5 cocoon; (**h**) CB-15 cocoon; (**i**) CB-12.5 cocoon. Scale bar: 100 μm.

**Figure 3:**
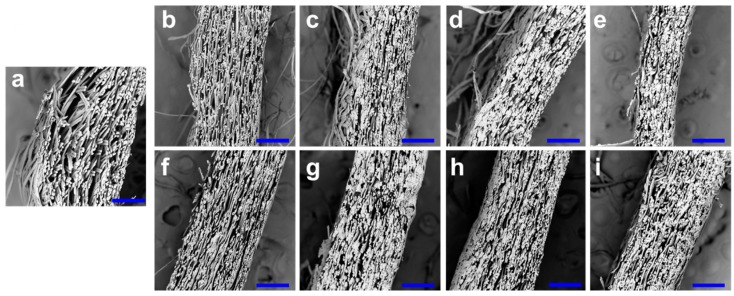
SEM images of cocoon cross-section structures. (**a**) Normal cocoon; (**b**) PT-25 cocoon; (**c**) PT-20 cocoon; (**d**) PT-14 cocoon; (**e**) PT-12 cocoon; (**f**) CB-20 cocoon; (**g**) CB-17.5 cocoon; (**h**) CB-15 cocoon; (**i**) CB-12.5 cocoon. Scale bar: 300 μm.

**Figure 4:**
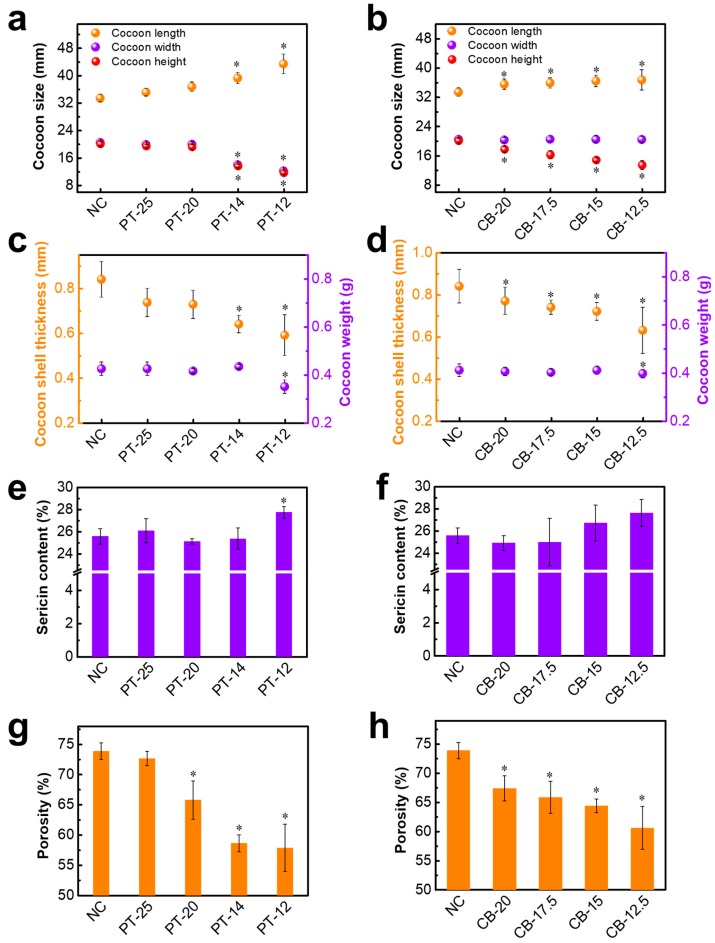
Parameters of silkworm cocoons. (**a**,**b**) Cocoon size; (**c**,**d**) cocoon thickness and weight; (**e**,**f**) cocoon sericin content; (**g**,**h**) cocoon porosity. Data are mean ± sd. Statistical analyses were performed using unpaired two-tailed Student’s *t* test (* *p* < 0.05).

Photographs (Figure 1) and SEM images (Figure 2 and Figure 3) of PT and CB cocoons clearly show the comparison between their morphologies and microstructures. These images reveal that different cocooning shapes or spaces lead to different cocoon morphologies and microstructures. By controlling the cocooning shape or space, the silkworm cocoons are shaped according to the given spinning tool (Figure 1). PT cocoons were spun by silkworms in paper tubes with different diameters, while CB cocoons were obtained from cardboard boxes with different widths. Figure 4a,b show the size (including length, width, and height) of each cocoon. For PT cocoons, their width decreases and the length increases as the diameter of the paper tube decreases. Meanwhile, the length of CB cocoons increases, and the height of the cocoon decreases with the decrease in the box’s width.

Both PT and CB cocoons have similar nonwoven composite structures with multiple layers parallel to the surface direction (see SEM images in Figure 2 and Figure 3). Compared to the NCs, the PT and CB cocoons are more compact and have denser fiber networks. Figure 4c shows that the shell thickness of PT cocoons decreases from 0.74 to 0.59 mm with the decrease in paper tube diameter. The cocoon shell thickness of CB cocoons changes in a similar way. Figure 4d indicates that there is no significant difference between the weight of normal cocoons and the PT and CB cocoons, except for PT-12. This means that decreasing the cocooning space to a certain limit does not change the number of silk fibers produced by silkworm larvae. When the cocooning space is smaller than the size limit, silkworm larvae are in a state of subhealth and cannot finish the cocooning process, resulting in a reduction in the number of silk fibers.

In addition, solidification contraction of silk fibers occurs when they are spun from the spinneret to the air during the construction of the cocoon frame, resulting in a rough cocoon surface. The sericin coating in the outer layer of the cocoon does not interconnect the fibers, so it does not form additional bonding between fibers. In contrast, for cocoons prepared from a confined cocooning space, there is not enough room for silkworm larvae to build a capacious cocoon frame. The fibers are confined and bonded together in a relatively narrow space. This method avoids the contraction of the surface layer and can form a smooth cocoon surface and a highly bonded network. Overall, the modified cocoons whose shapes are in accordance with the given cocooning space are constructed without any negative influence on cocoon weight. Both PT and CB cocoons show a denser structure and better homogeneity compared to the normal ones (shown in Figure 2 and Figure 3). The homogeneity of the layer structure of most of the modified cocoons is superior to that of the normal cocoons and may contribute to the enhanced mechanical performance of these modified cocoons.

### 3.2. Sericin Content

Comparison between the sericin content of PT cocoons and CB cocoons was carried out and is shown in Figure 4e,f. It can be observed that the average sericin content of NCs is about 26%, which matches well with the results in previous studies [10]. Most of the modified cocoons, except PT-12, do not exhibit a significant increase or decrease in the sericin content compared to NCs, indicating that the decrease in cocooning space, to some extent, has little effect on the sericin content of silk fibers. However, there is a significant increase in the sericin content of PT-12. Combining the results of cocoon weight in Figure 4c with the sericin content of PT-12, the significant increase in the sericin content of PT-12 can be explained. Silkworm larvae in an excessively confined cocooning space cannot freely move their bodies and finish the cocooning process, which causes the lower weight of PT-12. The cocoon shell of PT-12 is speculated to be mainly made up of the silk fibers produced by silkworm larvae during the beginning of the cocooning process, and these initial fibers correspond to the outer layer of the normal silkworm cocoons. Furthermore, the test results by Kaur et al. [10] indicate that the outer layer of the normal cocoons has a higher sericin content compared to the other layers. Therefore, the higher sericin content of PT-12 can be well explained by this information.

### 3.3. Porosity of Cocoon Shells

To better understand the inner structure of these cocoons, cocoon shell porosity was calculated and averaged from six cocoon samples for each group, as shown in Figure 4g,h. The NC has a high porosity because there is a great deal of inter-fiber and interlayer free space [1,2,3,18]. However, the porosities of the PT-20, PT-14, and PT-12 cocoons were only 66%, 59%, and 58%, respectively, which are much lower than that of the NC (73%). The porosity of CB cocoons also decreases sharply with decreasing cocoon width, changing from 74% to 61%. The lower porosity of PT cocoons and CB cocoons is consistent with the microstructural observations shown in Figure 2 and Figure 3. The decrease in porosity of the two cocoons was achieved by the restriction of the cocooning space, which causes a decrease in interlayer distance. Silkworm cocoons have been proved to be structural property-dependent biocomposites from both experimental and theoretical results [1]. A tough and strong cocoon can be obtained through the design of the microstructure and with the effective introduction of stronger inter-fiber bonding [18]. The study by Guan et al. [18] indicates that the silk fiber network plays an important role in cocoon properties. Thus, the relatively lower porosity of the modified silkworm cocoons in this study could be beneficial to the formation of robust fiber networks and lead to enhanced tensile and peeling properties.

### 3.4. Mechanical Properties of Cocoon Specimens

**Figure 5:**
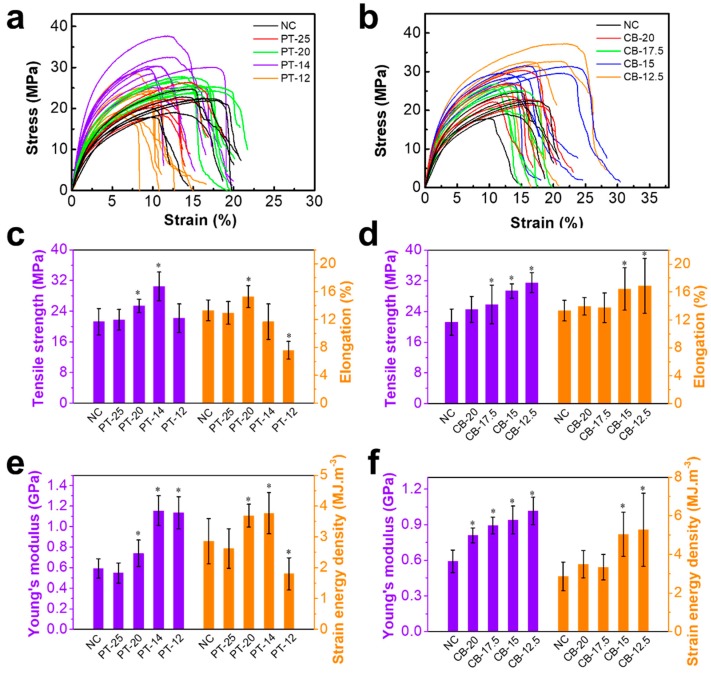
Mechanical properties of each type of silkworm cocoon. (**a**,**b**) Stress–strain curves; (**c**,**d**) tensile strength and elongation; (**e**,**f**) Young’s modulus and strain energy density. Data are mean ± sd. Statistical analyses were performed using unpaired two-tailed Student’s *t* test (* *p* < 0.05).

The tensile stress–strain curves and the tensile strength, elongation, Young’s modulus, and strain energy density (calculated from the accumulated stress–strain area prior to maximum stress) from different cocoon shell specimens are given in Figure 5. The stress–strain curves of PT cocoons and CB cocoons exhibit a similar trend with the decrease in cocooning space. The tensile strength, Young’s modulus, and strain energy density of both types of cocoons gradually increase. Particularly, PT-25 has similar mechanical properties to the NC. The PT-14 cocoon shows considerably improved Young’s modulus, tensile strength, and toughness with values of 1.2 GPa, 30.5 MPa, and 3.8 MJ/m^3^, which are much higher than those of the NC: 0.6 GPa, 21.2 MPa, and 2.9 MJ/m^3^. CB-12.5 is the most compact and solid in structure, resulting in the highest tensile strength, Young’s modulus, and strain energy density, which are increased by 66.7%, 48.6%, and 82.8%, respectively, compared to those of the NC. Nevertheless, when the cocooning space was smaller than the size limit (such as PT-12), silkworm larvae can no longer build a robust cocoon. This can be explained by the fact that too small a cocooning space limits the movement of the silkworm body and hinders silkworm cocoon formation, which finally leads to the degradation of the mechanical properties of the cocoon.

**Figure 6:**
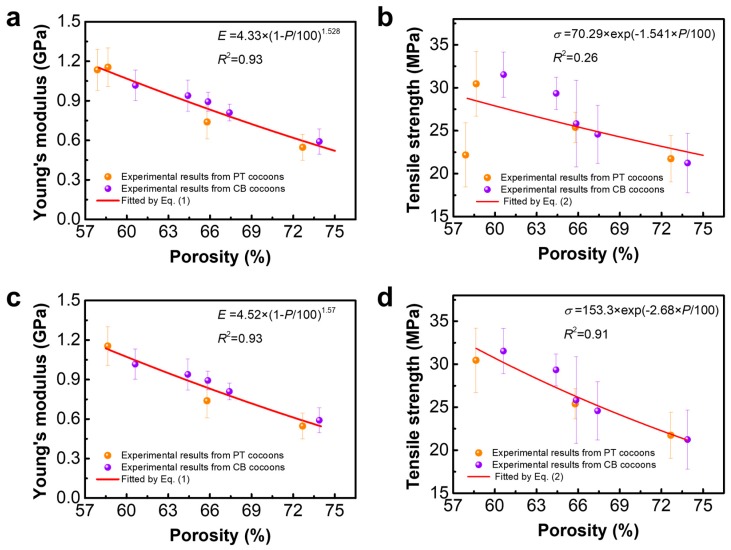
Porosity dependence of (**a**) Young’s modulus and (**b**) tensile strength for all the silkworm cocoons; porosity dependence of (**c**) Young’s modulus and (**d**) tensile strength for the silkworm cocoons without PT-12.

The enhanced mechanical properties are mainly due to the denser layer structure of the modified silkworm cocoons. The porosity should especially be integrated into the evaluation of composite mechanical performance [46,47,48]. The porosity dependence of Young’s modulus or tensile strength for silkworm cocoons is shown in Figure 6. Young’s modulus of silkworm cocoons increases from 0.5 GPa to 1.2 GPa with a decrease in porosity from 74% to 58%, while the tensile strength increases from 21 MPa to 32 MPa. Then, the porosity-dependent Young’s modulus (Figure 6a) and tensile strength (Figure 6b) of silkworm cocoons were fitted by Equations (1) and (2), respectively. A high goodness of fit of Young’s modulus (R^2^ = 0.93) was obtained, which indicates that Young’s modulus of the cocoons follows an exponential increase with decreasing porosity. However, the goodness of fit of tensile strength (R^2^ = 0.26) is quite low. Notably, the tensile strength of PT-12 is far from having a good fitting result. According to the low weight and higher sericin content of PT-12, we can find that silkworm larvae in an excessively confined cocooning space cannot freely move their bodies and finish the cocooning process, and therefore, PT-12 is not a complete cocoon. It may no longer follow the variation tendency of the tensile strength of other PT cocoons. To verify this, we present the fitting results without the PT-12 values of Young’s modulus and tensile strength in Figure 6c,d. A high goodness of fit for Young’s modulus (R^2^ = 0.93) and tensile strength (R^2^ = 0.91) is obtained. This result indicates that the mechanical properties of these silkworm cocoons, except for those of PT-12, follow an exponential relation. PT-12, which is not a complete cocoon and was constructed in very limited cocooning space, exhibits an anomalous low tensile strength, meaning that an excessively confined cocooning space has a negative effect on the tensile strengths of silkworm cocoons. For the other cocoon groups, the lower porosity is related to denser fiber networks and the larger bonding area between fibers. Then, as the fibers bonding increases, fibers are more prone to fracture, rather than unraveling from each other, which leads to the increase in Young’s modulus and tensile strength.

### 3.5. Interlaminar Peel Properties

**Figure 7:**
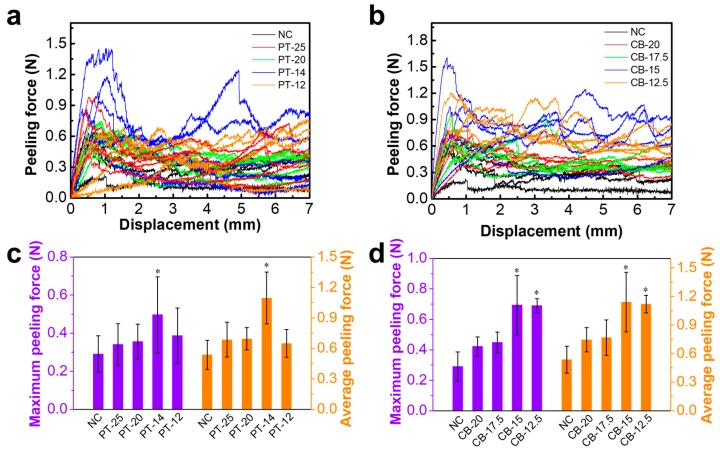
Comparison of peeling properties among different types of cocoons. (**a**–**b**) Peeling force curves of the cocoon specimens; (**c**–**d**) maximum peeling force and average peeling force. Data are mean ± sd. Statistical analyses were performed using unpaired two-tailed Student’s *t* test (* *p* < 0.05).

The peeling force-displacement relation, maximum peeling force, and average peeling force of PT cocoons and CB cocoons are shown in Figure 7. The interlayer connectivity in these cocoons has a similar nonlinear load-displacement relation (Figure 7a). The average and maximum peeling force of normal cocoons are 0.29 N and 0.54 N, respectively. The layer of the NC is easy to peel apart due to the uneven spatial distribution of sericin bonds and low interlaminar bonding [42]. PT cocoons exhibit relatively higher peel forces, with an average and maximum force of 0.34 N and 0.58 N for PT-25, 0.36 N and 0.69 N for PT-20, 0.50 N and 1.10 N for PT-14, and 0.39 N and 0.65 N for PT-12. The highest average and maximum force (PT-14 cocoon) are 1.72 times and 2.04 times higher than those of the control group. As for CB cocoons, their average peel forces increase from 0.29 N to 0.69 N while the maximum forces increase from 0.54 N to 1.14 N. The significant increase in the peel forces for PT and CB cocoons (except PT-12) indicates that high interlaminar bonding can be obtained using methods to control the cocooning space. The relatively weak interlaminar bonding in PT-12 indicates that too narrow a cocooning space has a negative effect on the peel resistance of silkworm cocoons. As discussed in the above sections, this could be explained by (1) the spinning motions of silkworm larvae are limited by too narrow a cocooning space; (2) the limited spinning motions increase the inhomogeneity and induce more flaws in the silk fiber networks. If the cocooning space is set just as the body size of the silkworm larvae, their cocooning process can hardly be completed. This case should be avoided. In summary, in a relatively narrow space, the silk fibers from silkworm larvae more easily bond together and the process can be regarded as a natural pressing process. It is reported that the peel load of silkworm cocoons obtained by hot pressing at room temperature is lower than that of normal silkworms, which indicates that the high-pressure compression process can destroy the interlayer bonding structure of the cocoon and lead to cracks in the silkworm cocoon [42]. However, the natural pressing process in this study does not damage their quality or interlayer bonding structure and provides silkworm cocoons with robust fiber networks and compact layer structures. More fiber bonds and a larger bonding area located in the compact layers are key contributors to the higher peel resistance of these modified cocoons.

## 4. Conclusions

In this study, we first fabricated a series of modified silkworm cocoons (PT cocoons and CB cocoons) with special shapes, dense structures, and excellent mechanical properties through a kind of natural pressing process. The microstructures, morphologies, compositions, and mechanical properties of these novel cocoons were characterized and compared with the normal silkworm cocoons. Most of these modified silkworm cocoons have denser fiber networks and lower porosities. Above a size limit of the cocooning space, the tensile strengths, Young’s moduli, and strain energy densities of these modified silkworm cocoons increase with the decrease in cocooning space. Notably, the tensile strength and Young’s modulus of the PT-14 cocoon are found to be 1.44 and 2 times higher than those of the normal cocoons, respectively. As expected, most of these modified silkworm cocoons also exhibit excellent peeling resistance. The strengthening mechanisms underlying the method for controlling the cocooning space are attributed to the densification of silkworm cocoons by a natural pressing process, which leads to a robust fiber network and improves the load capacity of these cocoons. These special cocoons with excellent mechanical properties could have potential applications in the production of high-performance artificial cocoon composites and biomimetic materials.
